# Analysis of risk factors for PICC-related venous thrombosis in patients with hematological malignancies and establishment of a nomogram prediction model

**DOI:** 10.3389/fonc.2026.1675200

**Published:** 2026-02-04

**Authors:** Xianzhi Zhao, Zhan Su, Zehua Wang, Wanting Sheng, Xiaojia Pu, Xiaoyun Yu, Shanshan Gao

**Affiliations:** 1 Department of Hematology, The Affiliated Hospital of Qingdao University, Qingdao, China; 2 Department of Orthopaedic Surgery, The Affiliated Hospital of Qingdao University, Qingdao, China

**Keywords:** catheter-related vein thrombosis, hematologic malignancies, nomogram, peripherally inserted central catheter, risk prediction

## Abstract

**Objective:**

To analyze the independent risk factors for PICC-related venous thrombosis in patients with hematologic malignancies, and to construct and validate a risk prediction model.

**Methods:**

This retrospective study analyzed data from 264 hematologic malignancy patients who received PICC chemotherapy at the Affiliated Hospital of Qingdao University between January 2022 and December 2024. Patients were randomly divided into training and validation sets (7:3), and the incidence of CRT was calculated. In the training set, LASSO regression and multivariable logistic regression identified independent CRT risk factors, which were used to construct a predictive nomogram. The model’s discrimination, calibration, and clinical utility were evaluated using AUC, calibration curves, and DCA.

**Results:**

The prevalence of PICC-related venous thrombosis was 6.1%, with 16 out of 264 patients diagnosed with CRT. Multivariable logistic regression analysis identified seven independent risk factors for CRT: hemoglobin, platelet count, prothrombin time, D-dimer, globulin, punctured vein, and catheter insertion depth. The area under the receiver operating characteristic curve for the training and validation sets was 0.965 and 0.977, respectively. Calibration and decision curve analyses demonstrated that the nomogram had good predictive accuracy and clinical utility in estimating CRT risk in patients with hematologic malignancies.

**Conclusions:**

In this study, we identified independent risk factors for CRT following PICC placement in patients with hematologic malignancies and developed a predictive model to assess CRT risk. The model demonstrated good discrimination, calibration, and clinical utility, enabling individualized risk assessment and intervention strategies to improve chemotherapy safety and extend PICC use duration.

## 1 Introduction

Hematologic malignancies are a group of clonal disorders arising from hematopoietic or lymphoid tissues and are broadly classified into three main categories: leukemia, lymphoma, and multiple myeloma (1, 2). These malignancies are characterized by genetic or epigenetic abnormalities occurring at various stages of hematopoietic cell differentiation, leading to the uncontrolled proliferation of abnormal cells in the bone marrow, peripheral blood, or lymphatic system. This clonal expansion disrupts normal hematopoiesis and immune function, resulting in clinical manifestations such as anemia, bleeding, infection, lymphadenopathy, hepatosplenomegaly, and bone pain (2). Among these, acute leukemia is noted for its rapid disease progression and typically necessitates prompt initiation of intensive chemotherapy (3), whereas lymphoma and multiple myeloma often require long-term, multi-cycle combination therapies, including chemotherapy, targeted agents, immunotherapy, and hematopoietic stem cell transplantation (4).

In recent years, advancements in precision medicine, immunotherapy, and molecular diagnostics have significantly deepened our understanding of the pathogenesis of hematologic malignancies. Correspondingly, treatment strategies have shifted from traditional chemotherapy to more individualized and combination-based approaches. Novel therapies, including targeted agents and immunotherapies such as CAR-T cell therapy, have increasingly become part of the standard of care, markedly improving the prognosis in certain patient populations (5, 6). However, these regimens are frequently associated with severe adverse effects, such as myelosuppression and immunosuppression, necessitating continuous and reliable intravenous access for the administration of chemotherapeutic agents, antibiotics, hematopoietic growth factors, nutritional support, and frequent blood sampling—demands that often exceed the capacity of conventional peripheral venous access (7). Furthermore, complications like thrombocytopenia and coagulation disorders substantially elevate the risks and technical difficulty associated with repeated venipuncture (8).

Peripherally inserted central catheter (PICC) is a safe, stable, and long-term venous access device that plays a critical role in the treatment of hematologic malignancies and is now widely used in clinical practice (9). PICC not only reduces the frequency of venous punctures during chemotherapy but also improves the safety and adherence of hypertonic drug infusions, thereby facilitating the smooth implementation of complex treatment regimens (10). Consequently, PICC has become an essential component in the comprehensive management of patients with hematologic malignancies, particularly those requiring prolonged chemotherapy, intensive infusion support, or who are at high risk of bleeding. However, as an invasive procedure, PICC can damage venous endothelium, and the infusion of chemotherapeutic agents may further irritate the vessel wall, increasing the risk of catheter-related thrombosis (CRT) after insertion (11). CRT not only compromises catheter function but may also lead to life-threatening complications, such as pulmonary embolism, if thrombi dislodge and migrate through the bloodstream (12). As a result, thromboprophylaxis following PICC placement in patients with hematologic malignancies has garnered increasing clinical attention. Despite this, few studies have comprehensively integrated the risk factors associated with CRT in this population to enable individualized prediction.

A nomogram, constructed from multivariable regression models, offers a visual and quantitative tool for estimating the probability of clinical outcomes and supports personalized decision-making. However, to date, no predictive model has been established for assessing CRT risk after PICC placement in patients with hematologic malignancies. Therefore, this study aims to identify independent risk factors for CRT in this population and to develop a nomogram capable of accurately predicting its occurrence, thereby providing a basis for informed clinical decision-making.

## 2 Methods

### 2.1 Study design and subject selection

**Figure 1:**
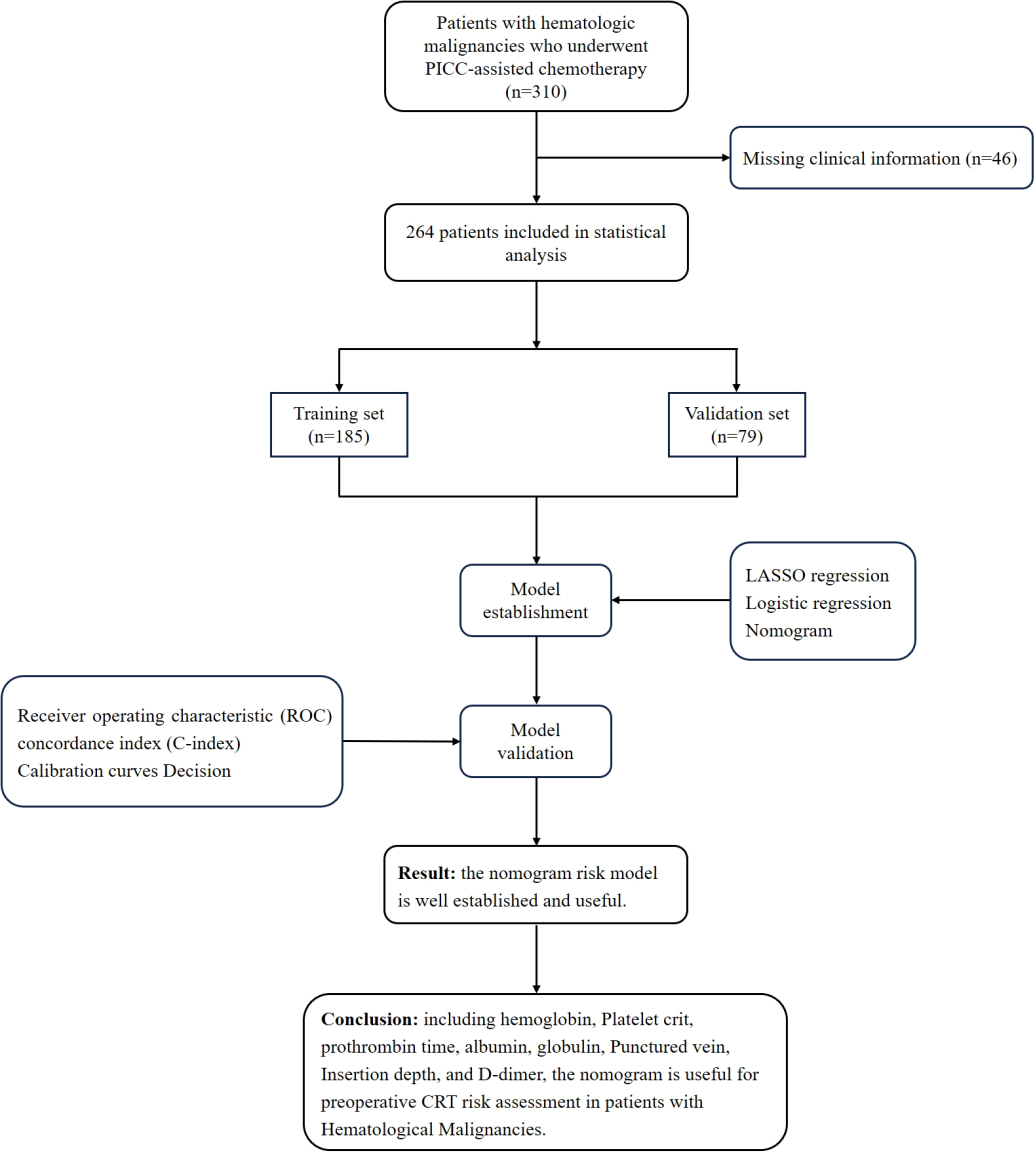
Flow diagram of study design.

Clinical data were retrospectively collected from the patient information database of the Affiliated Hospital of Qingdao University. A total of 310 patients with hematologic malignancies who underwent PICC-assisted chemotherapy in the Department of Hematology between January 2022 and December 2024 were initially identified. Among them, 46 patients were excluded due to incomplete clinical data, resulting in a final study cohort of 264 patients. CRT was diagnosed using Doppler ultrasonography. The diagnostic criteria included the presence of hypoechoic or hyperechoic intraluminal thrombus in the affected vein, partial or complete venous incompressibility, and absent or reduced blood flow signals (13). These patients were retrospectively analyzed and categorized into two groups based on the occurrence of CRT following PICC insertion: the thrombus group (n = 16) and the non-thrombus group (n = 248). Inclusion criteria were as follows: (1) diagnosis of hematologic malignancy (including leukemia, lymphoma, multiple myeloma, or myelodysplastic syndrome) confirmed by histopathology; (2) PICC insertion via the median cubital vein or basilic vein; (3) completion of venous color Doppler ultrasonography to screen for thrombosis; (4) receipt of at least one cycle of chemotherapy; and (5) complete demographic and medical record data. Exclusion criteria included: (1) history of bleeding disorders or anticoagulant use; (2) presence of solid tumors at other anatomical sites; (3) unilateral termination of chemotherapy; and (4) incomplete clinical or demographic data. (**Figure 1**) This study was approved by the Ethics Committee of the Affiliated Hospital of Qingdao University (Approval No.: QYFY-WZLL-30053).

### 2.2 Data collection

The dataset was randomly divided into a training set (n = 185) and a validation set (n = 79) at a ratio of 7:3. Clinical data were extracted and organized by two researchers using the hospital’s electronic medical record system. Based on previously published studies on post-PICC thrombosis (8, 14), we compiled a set of variables potentially associated with the development of CRT following PICC placement in patients with hematologic malignancies. These variables included clinical characteristics, laboratory indices, and catheter-related information. Clinical characteristics comprised malignancy type, sex, age, height, weight, smoking status, alcohol consumption history, history of surgery, hormone use, hypertension, diabetes mellitus, and previous thrombotic events. Laboratory indices included white blood cell count (3.5–9.5 × 10^9^/L), red blood cell count (4.3–5.8 × 10¹²/L), hemoglobin (130–175 g/L), platelet count (125–350 × 10^9^/L), prothrombin time (PT) (9.4–12.5 s), international normalized ratio (INR) (0.8–1.2), plasminogen activity (80%–200%), fibrinogen (2.38–4.98 g/L), activated partial thromboplastin time (APTT) (25.1–38.4 s), APTT ratio (0.86–1.30), thrombin time (10.3–16.6 s), D-dimer (0–500 ng/mL), albumin (40–55 g/L), globulin (20–40 g/L), albumin-to-globulin ratio (1.2–2.4), total bilirubin (0–26 μmol/L), direct bilirubin (0–8 μmol/L), indirect bilirubin (1.7–10.2 μmol/L), triglycerides (0–1.7 mmol/L), total cholesterol (0–5.20 mmol/L), and fasting blood glucose (3.9–6.1 mmol/L). Based on the institutional laboratory reference ranges, these indicators were categorized as normal or abnormal (15). All laboratory tests were performed within 24 hours before PICC placement. Catheter-related variables included history of PICC use, history of central venous catheterization, vein of catheter insertion, limb of insertion, catheter tip position, insertion depth, catheter type, and number of puncture attempts.

### 2.3 Statistical analysis

All statistical analyses were performed using R software (version 4.3.1, https://www.r-project.org/). Variables were categorized as continuous or categorical based on their data type. Continuous variables were presented as mean ± standard deviation (mean ± SD) and compared between groups using independent samples t-tests. Categorical variables were expressed as frequency (percentage) and analyzed using the chi-square test. To identify independent risk factors for CRT following PICC placement in patients with hematologic malignancies, a two-step modeling approach was applied. First, the least absolute shrinkage and selection operator (LASSO) regression was used for variable selection. The selected variables were then entered into a multivariable logistic regression model, with results reported as odds ratios (ORs) and 95% confidence intervals (CIs). A nomogram was subsequently constructed based on the identified independent predictors to estimate the risk of CRT. The model’s discriminative ability was assessed by plotting the receiver operating characteristic (ROC) curve and calculating the area under the curve (AUC). A calibration curve was also generated to evaluate the agreement between predicted and observed outcomes. In addition, the concordance index (C-index) was calculated to quantify overall predictive accuracy. Finally, decision curve analysis (DCA) was performed to evaluate the clinical utility and net benefit of the model.

## 3 Results

### 3.1 Baseline characteristics

A total of 264 patients with hematologic malignancies who underwent PICC placement were included in the study. Among them, 16 patients were diagnosed with CRT using venous color Doppler ultrasonography, yielding a prevalence of 6.1%. The baseline characteristics of all participants are summarized in **Table 1**. Patients were randomly assigned to training and validation sets in a 7:3 ratio. No statistically significant differences were observed between the two groups across all baseline variables (P > 0.05, **Table 2**).

**Table 1:** Characteristics of patients in the non-CRT group and CRT group.

Characteristics	Non-CRT group (n=248)	CRT group (n=16)	P-value
Age	52.12 ± 16.56	55.19 ± 19.97	0.479
BMI	24.15 ± 3.81	23.34 ± 3.28	0.408
Insertion depth	40.43 ± 2.91	38.50 ± 2.92	0.011
Number of puncture attempts	0.01 ± 0.13	0.13 ± 0.34	<0.001
Malignancy type			0.224
Leukemia	106 (42.7%)	3 (18.8%)	
Lymphoma	117 (47.2%)	10 (62.5%)	
Myeloma	20 (8.1%)	2 (12.5%)	
MDS	5 (2%)	1 (6.3%)	
Gender			0.727
Male	144 (58.1%)	10 (62.5%)	
Female	104 (41.9%)	6 (37.5%)	
Smoking history			0.504
Yes	32 (12.9%)	3 (18.8%)	
No	216 (87.1%)	13 (81.3%)	
Alcohol consumption history			0.574
Yes	34 (13.7%)	3 (18.8%)	
No	214 (86.3%)	13 (81.3%)	
Surgery history			0.590
Yes	77 (31%)	6 (37.5%)	
No	171 (69%)	10 (62.5%)	
Hormone use			0.742
Yes	119 (48%)	7 (43.8%)	
No	129 (52%)	9 (56.3%)	
Hypertension			0.258
Yes	75 (30.2%)	7 (43.8%)	
No	173 (69.8%)	9 (56.3%)	
Diabetes			0.672
Yes	41 (16.5%)	2 (12.5%)	
No	207 (83.5%)	14 (87.5%)	
Chemotherapy history			0.213
Yes	101 (40.7%)	4 (25%)	
No	147 (59.3%)	12 (75%)	
WBC			0.614
Abnormal	109 (44%)	6 (37.5%)	
Normal	139 (56%)	10 (62.5%)	
RBC			0.953
Abnormal	200 (80.6%)	13 (80.7%)	
Normal	48 (19.4%)	3 (18.3)	
HB			0.33
Abnormal	209 (84.3%)	12 (75%)	
Normal	39 (15.7%)	4 (25%)	
PLT			0.164
Abnormal	122 (49.2%)	5 (31.3%)	
Normal	126 (50.8%)	11 (68.8%)	
PTs			0.007
Abnormal	74 (29.8%)	10 (62.5%)	
Normal	174 (70.2%)	6 (37.5%)	
PT%			0.475
Abnormal	58 (23.4&)	5 (31.3%)	
Normal	190 (76.6%)	11 (68.8%)	
INR			0.333
Abnormal	52 (21%)	5 (31.3%)	
Normal	196 (79%)	11 (68.8%)	
FIB			0.45
Abnormal	57 (23%)	5 (31.3%)	
Normal	191 (77%)	11 (68.8%)	
APTT			0.919
Abnormal	14 (5.6%)	1 (6.3%)	
Normal	234 (94.4%)	15 (93.8%)	
TTs			0.506
Abnormal	29 (11.7%)	1 (6.3%)	
Normal	219 (88.3%)	15 (93.8)	
D-dimer			0.003
Abnormal	121 (48.8%)	14 (87.5%)	
Normal	127 (51.2%)	2 (12.5%)	
ALB			0.035
Abnormal	152 (61.3%)	14 (87.5%)	
Normal	96 (38.7%)	2 (12.5%)	
GLB			0.006
Abnormal	62 (25%)	9 (56.3%)	
Normal	186 (75%)	7 (43.8%)	
A/G			0.823
Abnormal	56 (22.6%)	4 (25%)	
Normal	192 (77.4%)	12 (75%)	
TBIL			0.355
Abnormal	53 (21.4%)	5 (31.3%)	
Normal	195 (78.6%)	11 (68.8%)	
DBIL			0.891
Abnormal	34 (13.7%)	2 (12.5%)	
Normal	214 (86.3%)	14 (87.5%)	
IBIL			0.295
Abnormal	16 (6.5%)	0 (0%)	
Normal	232 (93.5%)	16 (100%)	
GLU			0.705
Abnormal	73 (29.4%)	4 (25%)	
Normal	175 (70.6%)	12 (75%)	
TG			0.566
Abnormal	76 (30.6%)	6 (37.5%)	
Normal	172 (69.4%)	10 (62.5)	
TC			0.416
Abnormal	52 (21%)	2 (12.5%)	
Normal	196 (79%)	14 (87.5%)	
Punctured vein			0.002
Basilic vein	235 (94.8%)	12 (75%)	
Brachial vein	13 (5.2%)	4 (25%)	
Catheter side			0.987
Left	77 (31%)	5 (31.3%)	
Right	171 (69%)	11 (68.8)	
History of central venous catheterization			0.427
Yes	70 (28.2%)	6 (37.5%)	
No	178 (71.8%)	10 (62.5%)	
Catheter type			0.414
4F	33 (13.3%)	1 (6.3%)	
5F	215 (86.7%)	15 (93.8%)	
PICC history of blood transfusion			0.805
Yes	85 (34.3%)	5 (31.3%)	
No	163 (65.7%)	11 (68.8%)	

BMI, Body mass index; PIR, Poverty income ratio; WBC, White blood cell; HGB, Hemoglobin; RBC, Red blood cell.

**Table 2:** Characteristics of training and validation sets.

Characteristics	Training set (n=185)	Validation set (n=79)	P-value
Age	52.5 ± 16.56	51.85 ± 17.32	0.960
BMI	23.89 ± 4	24.6 ± 3.2	0.071
Insertion depth	40.28 ± 2.74	40.39 ± 3.38	0.975
Number of puncture attempts	0.02 ± 0.15	0.03 ± 0.16	0.854
Malignancy type			0.313
Leukemia	72 (38.9%)	37 (46.8%)	
Lymphoma	90 (48.6%)	37 (46.8%)	
Myeloma	17 (9.2%)	5 (6.3%)	
MDS	6 (3.2%)	0 (0%)	
Gender			1
Male	108 (58.4%)	46 (58.2%)	
Female	77 (41.6%)	33 (41.8%)	
Smoking history			0.684
Yes	23 (12.4%)	12 (15.2%)	
No	162 (87.6%)	67 (84.8%)	
Alcohol consumption history			0.868
Yes	25 (13.5%)	12 (15.2%)	
No	160 (86.5%)	67 (84.8%)	
Surgery history			0.699
Yes	60 (32.4%)	23 (29.1%)	
No	125 (67.6%)	56 (70.9%)	
Hormone use			0.831
Yes	87 (47%)	39 (49.4%)	
No	98 (53%)	40 (50.6%)	
Hypertension			0.569
Yes	55 (29.7%)	27 (34.2%)	
No	130 (70.3%)	52 (65.8%)	
Diabetes			0.552
Yes	28 (15.1%)	15 (19%)	
No	157 (84.9%)	64 (81%)	
Chemotherapy history			1
Yes	74 (40%)	31 (39.2%)	
No	111 (60%)	48 (60.8%)	
WBC			0.805
Abnormal	82 (44.3%)	33 (41.8%)	
Normal	103 (55.7%)	46 (58.2%)	
RBC			1
Abnormal	149 (80.5%)	64 (81%)	
Normal	36 (19.5%)	15 (19%)	
HB			1
Abnormal	155 (83.8%)	66 (83.5%)	
Normal	30 (16.2%)	13 (16.5%)	
PLT			1
Abnormal	86 (46.5%)	41 (51.9%)	
Normal	99 (53.5%)	38 (48.1%)	
PTs			1
Abnormal	59 (31.9%)	25 (31.6%)	
Normal	126 (68.1%)	54 (68.4%)	
PT%			0.404
Abnormal	41 (22.2%)	22 (27.8%)	
Normal	144 (77.8%)	57 (72.2%)	
INR			0.261
Abnormal	36 (19.5%)	21 (26.6%)	
Normal	149 (80.5%)	58 (73.4%)	
FIB			0.211
Abnormal	39 (21.1%)	23 (29.1%)	
Normal	146 (78.9%)	56 (70.9%)	
APTT			0.775
Abnormal	10 (5.4%)	5 (6.3%)	
Normal	175 (94.6%)	74 (93.7%)	
TTs			0.532
Abnormal	23 (12.4%)	7 (8.9%)	
Normal	162 (87.6%)	72 (91.1%)	
D-dimer			0.809
Abnormal	96 (51.9%)	39 (49.4%)	
Normal	89 (48.1%)	40 (50.6%)	
ALB			0.818
Abnormal	115 (62.2%)	51 (64.6%)	
Normal	70 (37.8%)	28 (35.4%)	
GLB			0.939
Abnormal	49 (26.5%)	22 (27.8%)	
Normal	136 (73.5%)	57 (72.2%)	
A/G			0.414
Abnormal	39 (21.1%)	21 (26.6%)	
Normal	146 (78.9%)	58 (73.4%)	
TBIL			0.781
Abnormal	42 (22.7%)	16 (20.3%)	
Normal	143 (77.3%)	63 (79.7%)	
DBIL			1
Abnormal	25 (13.5%)	11 (13.9%)	
Normal	160 (86.5%)	68 (86.1%)	
IBIL			0.784
Abnormal	12 (6.5%)	4 (5.1%)	
Normal	173 (93.5%)	75 (94.9%)	
GLU			0.306
Abnormal	135 (73%)	27 (34.2%)	
Normal	50 (27%)	52 (65.8%)	
TG			0.390
Abnormal	54 (29.2%)	28 (35.4%)	
Normal	131 (70.8%)	51 (64.6%)	
TC			0.435
Abnormal	35 (18.9%)	19 (24.1%)	
Normal	150 (81.1%)	60 (75.9%)	
Punctured vein			0.439
Basilic vein	175 (94.6%)	72 (91.1%)	
Brachial vein	10 (5.4%)	7 (8.9%)	
Catheter side			0.893
Left	57 (30.8%)	25 (31.6%)	
Right	128 (69.2%)	54 (68.4%)	
History of central venous catheterization			0.712
Yes	55 (29.7%)	21 (26.6%)	
No	130 (70.3%)	58 (73.4%)	
Catheter type			0.712
4F	22 (11.9%)	21 (26.6%)	
5F	163 (88.1%)	67 (84.8%)	
PICC history of blood transfusion			0.657
Yes	61 (33%)	29 (36.7%)	
No	124 (67%)	50 (63.3%)	

BMI, Body mass index; PIR, Poverty income ratio; WBC, White blood cell; HGB, Hemoglobin; RBC, Red blood cell.

### 3.2 LASSO regression analysis and multifactorial logistic regression analysis of the occurrence of CRT after PICC in patients with hematologic malignancies in the training set

**Figure 2:**
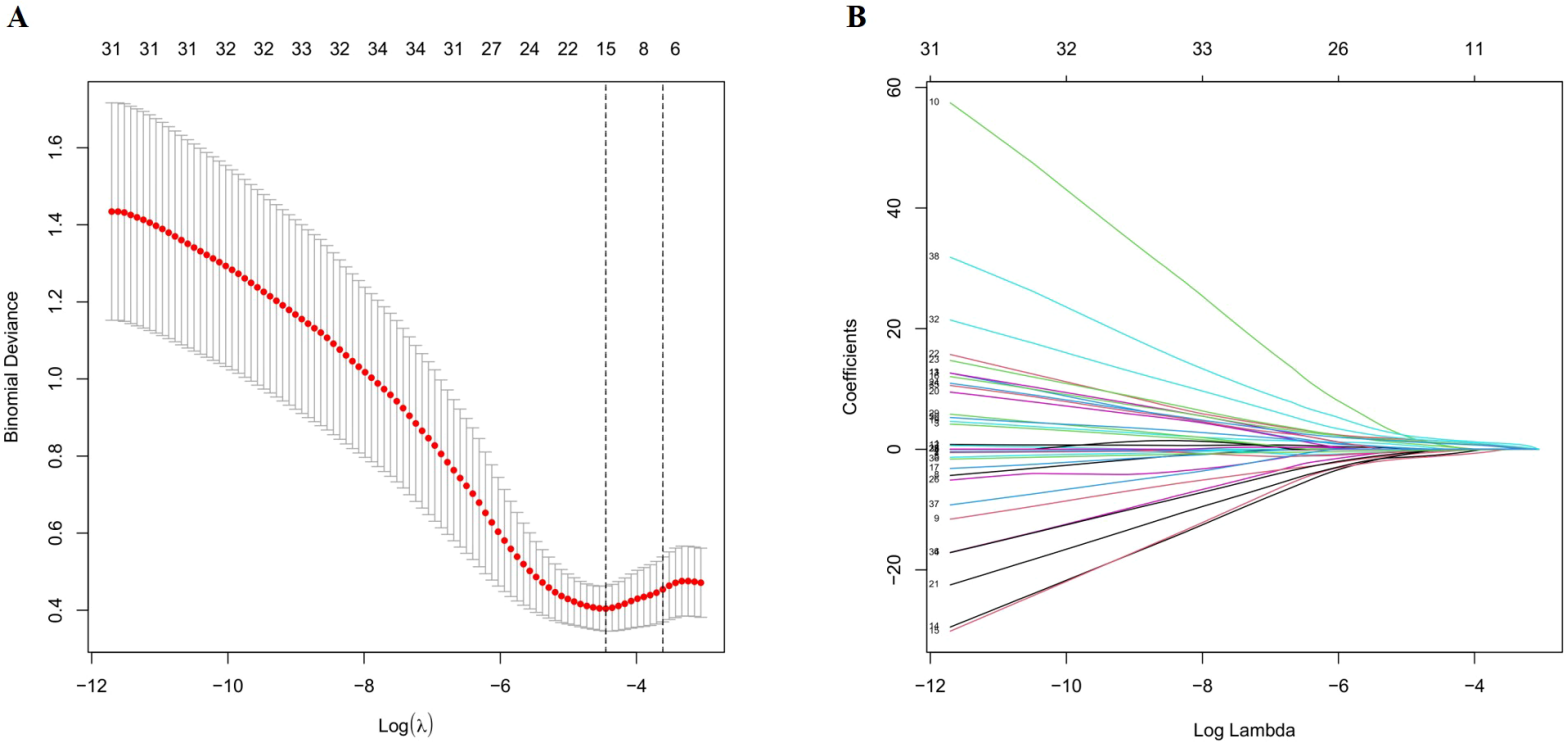
Selection of demographic and clinical risk features using the LASSO Regression Model. **(A)** Cross-validation of the LASSO model was performed using the minimum criterion, with dashed vertical lines indicating the optimal values (10 factors). **(B)** The 31 feature LASSO coefficient profiles for logarithmic (lambda) sequences are constructed.

Independent risk factors for CRT following PICC placement in patients with hematologic malignancies were identified using least absolute shrinkage and selection operator (LASSO) regression analysis on the training set. Twelve candidate variables were initially selected, including disease type, hypertension, hemoglobin (HGB), platelet count (PLT), prothrombin time (PTs), D-dimer, albumin (ALB), globulin (GLB), puncture vein, catheter insertion depth, number of puncture attempts, and others. Variable selection was optimized using 10-fold cross-validation (**Figure 2**). These 10 variables were then entered into a multivariable logistic regression model, with CRT occurrence as the dependent variable. The analysis identified seven independent predictors of CRT: HGB, PLT, PTs, D-dimer, GLB, punctured vein, and catheter insertion depth (**Table 3**).

**Table 3:** Factors associated with the risk of CRT in patients with hematological malignancies.

Intercept and variable	Assessment model		
	*β*	Odds ratio (95% CI)	P-value
Insertion depth	-0.361	0.697 (0.501-0.905	0.014
Number of puncture attempts	1.879	6.547 (0.115-302.61)	0.346
Malignancy type			
Leukemia			
Lymphoma	-0.262	0.769 (0.063-9.311)	0.832
Myeloma	-0.004	0.996 (0.030-24.968)	0.998
MDS	2.389	10.905 (0.244-683.666)	0.218
Hypertension			
Yes		1	
No	-0.606	0.545 (0.096-3.216)	0.489
HB			
Normal		1	
Abnormal	-3.858	47.40 (4.447-824.721)	0.003
PLT			
Normal		1	
Abnormal	-2.827	16.897 (2.453-197.994)	0.01
PTs			
Abnormal		1	
Normal	-2.503	0.082 (0.009-0.460)	0.01
D-dimer			
Abnormal		1	
Normal	-3.338	0.035 (0.002-0.288)	0.008
GLB			
Abnormal		1	
Normal	-1.947	0.143 (0.022-0.705)	0.022
Punctured vein			
Basilic vein		1	
Brachial vein	2.618	13.708 (1.214-176.031)	0.033

*β* is the regression coefficient.

CI, Confidence interval; WBC, White blood cell; HGB, Hemoglobin; RBC, Red blood cell.

### 3.3 Construction of a nomogram for the risk of CRT after PICC in patients with hematologic malignancies

**Figure 3:**
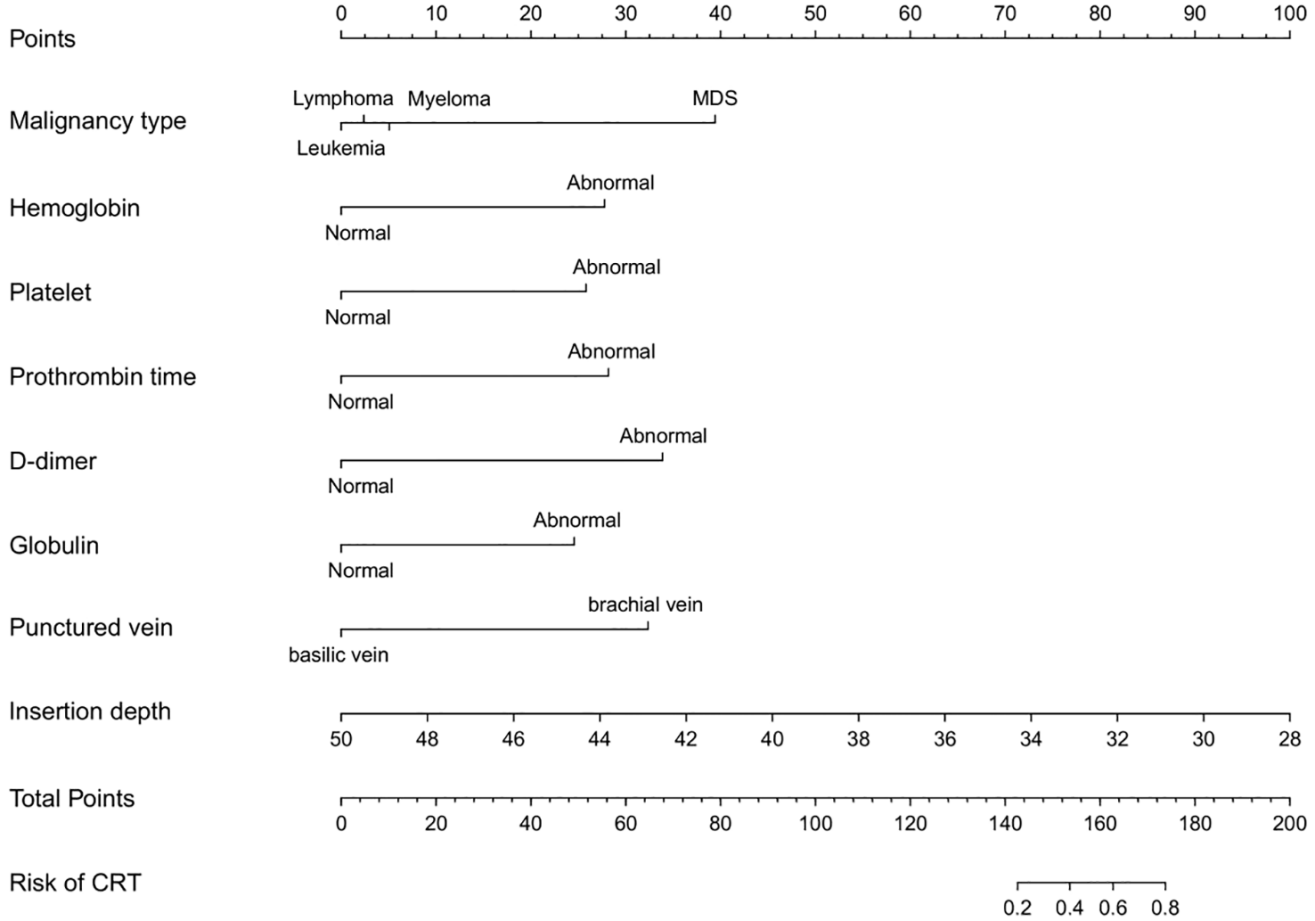
Development of a nomogram for predicting CRT risk in patients with hematological malignancies.

Although disease type did not reach statistical significance in the multivariable logistic regression analysis, it was retained in the nomogram because of its clinical relevance and biological plausibility. Prior studies have shown that thrombotic risk varies across malignancy subtypes and cancer types (16, 17), supporting the inclusion of disease-related characteristics. This approach improves the interpretability and clinical applicability of the model. Accordingly, a nomogram was developed to predict the risk of CRT following PICC placement in patients with hematologic malignancies, incorporating disease type along with the seven independent risk factors identified through multivariable logistic regression (**Figure 3**).

### 3.4 Evaluation of the effectiveness of the nomogram

**Figure 4:**
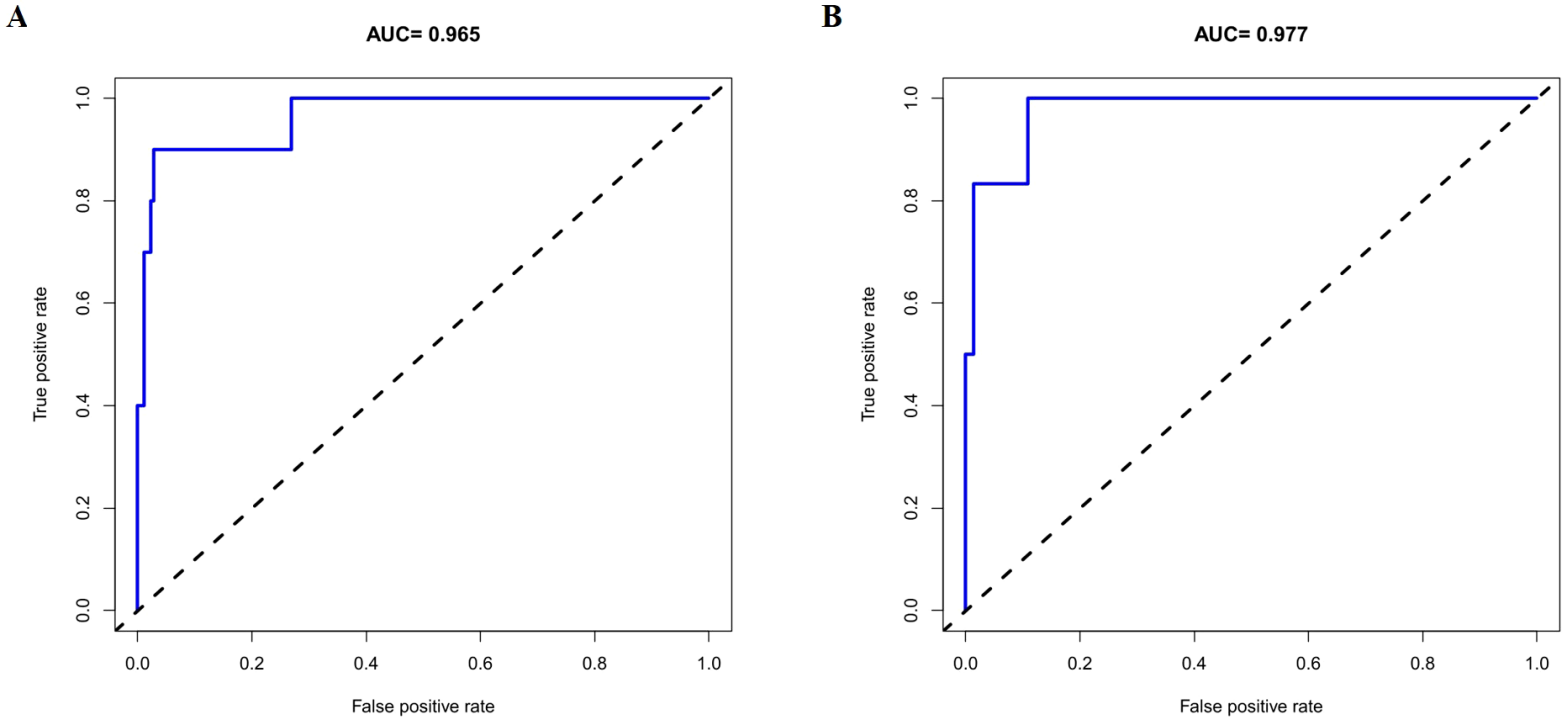
Receiver-operating characteristic (ROC) curves for predicting CRT among patients with hematological malignancies in training **(A)** and validation **(B)** sets.

**Figure 5:**
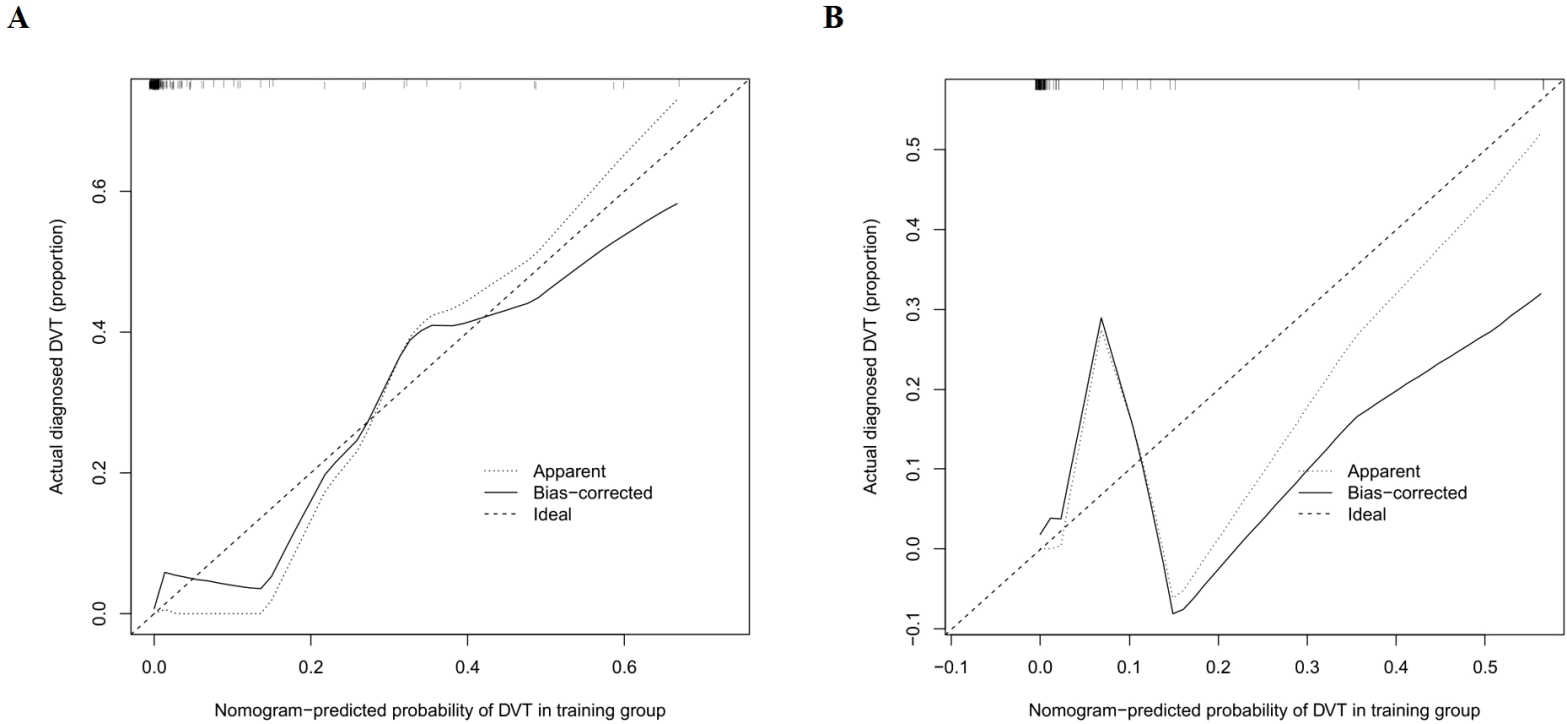
Calibration curves for the CRT risk nomogram in the training **(A)** and validation **(B)** sets.

**Figure 6:**
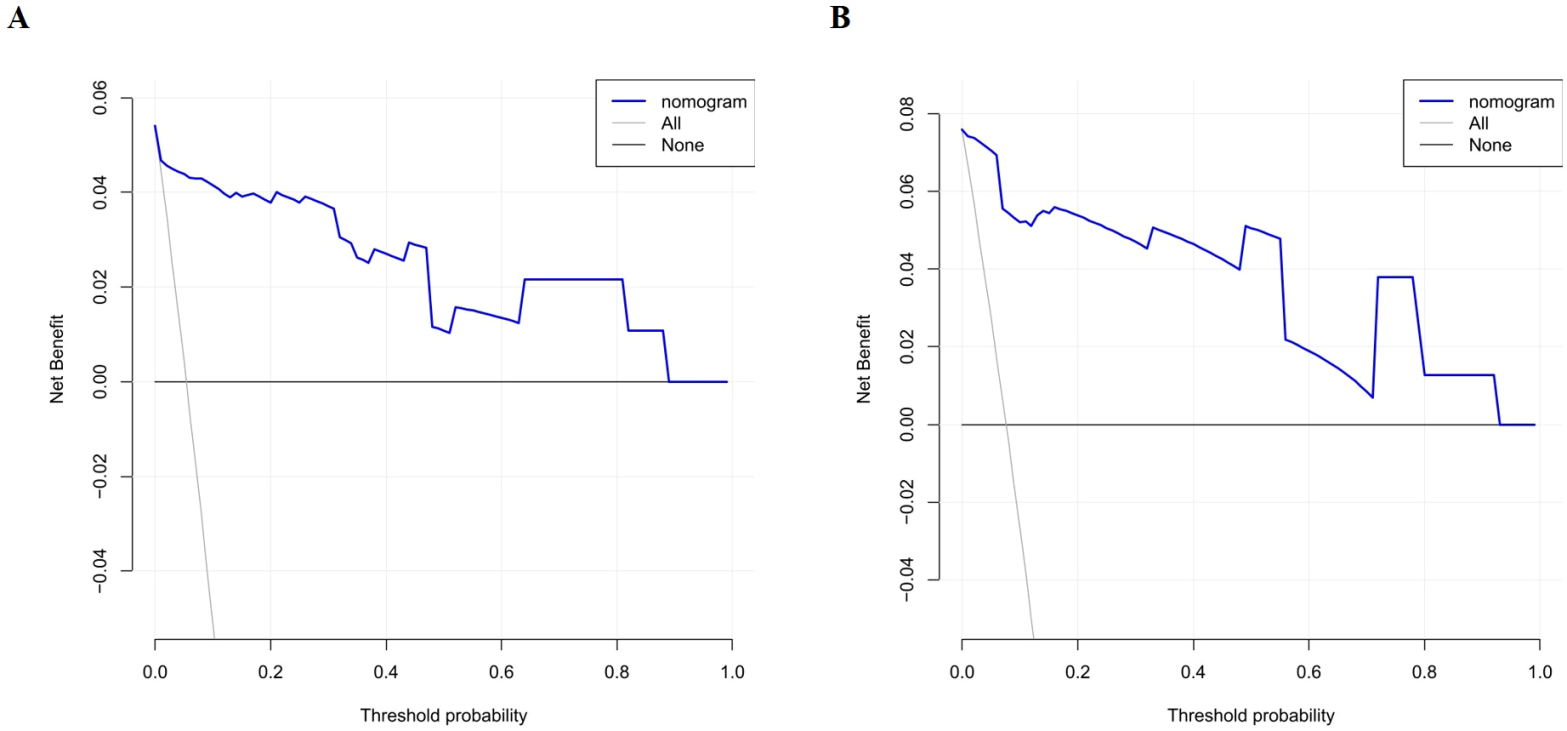
Decision curve analysis for the CRT risk nomogram in the training **(A)** and validation **(B)** sets.

The discriminative performance of the constructed nomogram was evaluated using receiver operating characteristic (ROC) curve analysis and the corresponding area under the curve (AUC). The AUCs for the training and validation sets were 0.965 (95% CI: 0.912 - 1) and 0.977 (95% CI: 0.939 - 1), respectively, indicating strong discriminatory ability (**Figure 4**). Calibration curves were plotted to assess the agreement between predicted probabilities and actual outcomes. Both calibration curves closely approximated the ideal 45-degree line, demonstrating good model calibration (**Figure 5**). Furthermore, clinical utility was evaluated using decision curve analysis (DCA). The model yielded high net benefits in both training and validation sets, supporting its practical value in predicting the risk of PICC-related CRT in patients with hematologic malignancies (**Figure 6**).

## 4 Discussion

Peripherally inserted central catheter has significant clinical utility in patients with hematologic malignancies (7). These patients often undergo high-intensity, multi-cycle chemotherapy, targeted therapy, or immunotherapy, with prolonged treatment durations and frequent intravenous drug administration. Complications such as bone marrow suppression and thrombocytopenia further increase the difficulty of using traditional peripheral venous access, which is frequently associated with puncture failure, vasculitis, and drug extravasation (7). PICC, inserted via an upper arm vein and advanced to the central vein under ultrasound guidance, offers advantages including ease of placement, extended maintenance duration, and broad applicability, making it a preferred option for intravenous therapy in this patient population (18). While PICC improves treatment adherence and quality of life, its associated complications—particularly CRT—should not be overlooked. CRT not only leads to catheter dysfunction and treatment interruption but may also cause severe outcomes such as pulmonary embolism, posing a serious threat to patient prognosis and treatment continuity (11). Therefore, the need for early identification and risk stratification of CRT has become increasingly urgent. However, there is currently no available risk assessment tool specifically designed to predict CRT in patients with hematologic malignancies following PICC placement, limiting the implementation of individualized prevention and intervention strategies. To address this gap, we retrospectively analyzed the clinical data of patients with hematologic malignancies who underwent PICC placement in our hospital over the past three years. We identified independent risk factors for CRT and developed a predictive model to facilitate early detection and personalized management of high-risk patients, ultimately aiming to enhance the safety and therapeutic effectiveness of PICC use.

In this study, the incidence of CRT following PICC placement in patients with hematologic malignancies was 6.1%, which is slightly lower than that reported by Yue et al. In their analysis of 117 patients with hematologic malignancies who underwent PICC placement, CRT occurred in 19 cases, yielding an incidence rate of 16.2% (7). Similarly, Wang et al. investigated 305 patients with lymphoma and reported a PICC-related venous thrombosis incidence of 11.48% (14). These discrepancies may be attributed to differences in demographic characteristics, medical history, baseline clinical conditions, catheter insertion and maintenance protocols, diagnostic criteria for CRT, and follow-up duration.

Numerous studies have demonstrated a significant association between hemoglobin concentration and the risk of thromboembolism (19). Malin Hultcrantz et al. analyzed data from 1.5 million blood donors and found that elevated hemoglobin levels were significantly correlated with an increased risk of arterial thrombosis, while low hemoglobin levels were associated with a higher risk of deep vein thrombosis (20). Elevated hemoglobin increases blood viscosity and slows blood flow, promoting a hypercoagulable state that favors thrombus formation. Conversely, decreased hemoglobin can activate plasma procoagulant factors and enhance platelet activity, indirectly contributing to thrombosis. Platelets and prothrombin time (PT) are critical indicators of coagulation status. Platelets are anucleate cellular components that play a central role in hemostasis and thrombosis (21, 22). Upon vascular injury, they rapidly adhere to the damaged endothelium, become activated, and release procoagulant substances, thereby initiating the coagulation cascade and forming a platelet-rich thrombus to seal the wound (23). In patients with hematologic malignancies, platelet levels are often influenced by both the underlying disease and treatment modalities. These fluctuations may be closely associated with the development of CRT. Prothrombin time is a routinely used clinical assay to assess the functionality of the extrinsic and common coagulation pathways, reflecting the integrated activity of factors II, V, VII, and X. PT has been shown to be associated with CRT risk (24, 25). In hematologic malignancy patients, PT alterations are common and may reflect either bleeding tendencies or a hypercoagulable state, often resulting from the impact of the disease or chemotherapy-induced hepatic and coagulation factor dysfunction. D-dimer is a soluble fibrin degradation product commonly used as a biomarker for coagulation abnormalities and an indicator of intravascular thrombosis (26). In a retrospective cohort study, Daniela R. Anderson et al. examined the association between D-dimer levels and thrombosis risk in 61 patients with acute lymphoblastic leukemia. The study showed that the cumulative incidence of venous or arterial thrombosis within 100 days of diagnosis was 52.9% in patients with high D-dimer levels (≥4 µg/mL), compared to 13.8% in those with low to moderate levels (<4 µg/mL) (27). These findings are consistent with our results and may be attributed to the hypercoagulable state commonly seen in patients with hematologic malignancies. This prothrombotic condition may be exacerbated by chemotherapy, infections, and the use of intravenous catheters, all of which can contribute to elevated D-dimer levels and an increased risk of CRT. Globulin, a key plasma protein synthesized primarily in the liver, plays a vital role in immune defense and anti-infective responses (28). Studies have shown that complement and antibodies are critical mediators of thrombosis. Reduced blood flow can cause immunoglobulin M (IgM) to bind to FcμR and polymeric immunoglobulin receptor (pIgR), leading to endothelial activation and platelet recruitment. This process promotes IgG deposition and classical complement activation, thereby initiating a prothrombotic cycle (29). Based on these mechanisms, hypergammaglobulinemia—including elevated polyclonal or monoclonal immunoglobulins—has a biologically plausible association with thrombotic risk (30). Elevated serum globulin levels may increase blood viscosity and disrupt hemodynamic stability, thereby promoting thrombogenesis (31). Notably, globulin abnormalities are common in patients with hematologic malignancies. In this population, the underlying malignancy or immune reconstitution therapies may alter immunoglobulin production, resulting in polyclonal or monoclonal immunoglobulin excess. These changes can increase blood viscosity and perturb coagulation homeostasis.

The development of CRT is also strongly associated with catheter-related factors (32). In our study, the site of PICC placement emerged as a significant risk factor, with insertion into the brachial vein associated with a higher risk of CRT compared to the basilic (also referred to as “vital”) vein. This may be attributed to the larger diameter, straighter course, and faster blood flow of the basilic vein, which may help prevent pericatheter blood stasis and thus reduce thrombotic risk. However, a retrospective study involving medical-surgical inpatients and outpatients found a lower incidence of deep vein thrombosis (DVT) when PICC was placed in the brachial vein (33). This discrepancy highlights the need for further research to determine the most appropriate venous access sites for PICC placement. Our results also indicated that catheter insertion depth was significantly associated with CRT risk. Specifically, shallower catheter placement was linked to a higher incidence of thrombosis. This may be due to insufficient advancement of the catheter tip into the central vein, which can result in reduced blood flow velocity, catheter instability, and increased local endothelial irritation—conditions that collectively promote thrombosis.

In conclusion, this study identified independent risk factors for CRT following PICC placement in patients with hematologic malignancies and developed a nomogram to predict CRT risk. This tool enables personalized risk assessment and may assist clinicians in implementing targeted interventions to enhance treatment safety during chemotherapy. However, several limitations should be acknowledged. First, this single-center retrospective study had a relatively small sample size, with all data derived from a single cohort. This design may introduce selection bias and limit the generalizability of the findings. Second, only 16 CRT events occurred in the cohort, yet the predictive model included eight predictors, resulting in a low events-per-variable ratio. This increases the risk of overfitting and may inflate estimates of model discrimination, including the AUC. Third, this study did not comprehensively assess other treatment-related factors, such as the type and timing of chemotherapy regimens, including targeted therapies. Previous studies have demonstrated that variations in chemotherapy regimens and their administration timing can affect the patency of vascular access devices and, consequently, the risk of catheter-related complications (34). Finally, the predictive model developed in this study was validated only internally and lacked external validation across multiple centers. Therefore, future research should expand the sample size, incorporate detailed chemotherapy-related variables, and perform multicenter external validation to improve the model’s reliability and generalizability.

## 5 Conclusion

In this study, we identified independent risk factors for CRT following PICC placement in patients with hematologic malignancies and developed a nomogram to assess CRT risk. The model demonstrated good discrimination, calibration, and clinical utility. Clinically, it can be used to quantitatively evaluate CRT risk in patients undergoing chemotherapy via PICC and guide individualized treatment strategies to enhance chemotherapy safety and extend PICC longevity.

## Data Availability

The original contributions presented in the study are included in the article/supplementary material. Further inquiries can be directed to the corresponding authors.
